# Non-invasive imaging of implanted peritoneal carcinomatosis in mice using PET and bioluminescence imaging

**DOI:** 10.1186/s13550-015-0125-z

**Published:** 2015-09-04

**Authors:** J. Stollfuss, N. Landvogt, M. Abenstein, S. Ziegler, M. Schwaiger, R. Senekowitsch-Schmidtke, H. Wieder

**Affiliations:** Department of Radiology and Nuclear Medicine, Klinikum Memmingen, Memmingen, Germany; Department of Radiology, Technische Universität München, Munich, Germany; Centre for Radiology and Nuclear Medicine, Grevenbroich, Germany; Department of Nuclear Medicine, Klinikum rechts der Isar, Technische Universität München, Munich, Germany

**Keywords:** Small animal imaging, Bioluminescence imaging, PET, Peritoneal carcinomatosis

## Abstract

**Background:**

Non-invasive imaging of peritoneal carcinomatosis remains challenging. The aim of this study was to compare positron emission tomography (PET) and bioluminescence imaging (BLI) for the early detection of peritoneal carcinomatosis in a mouse model.

**Methods:**

Female nude mice were inoculated intraperitoneally with 1×10^7^ HSC45-M2-luc gastric cancer cells. The cells were stably transfected with the gene coding for firefly luciferase. Tumour development was monitored using PET and BLI and in two subgroups, on days 3 and 4 or on days 6 and 7 after tumour cell inoculation. Tumour nodules found on post mortem examination served as the reference standard for evaluating the images.

**Results:**

PET detected 58/82 lesions (sensitivity 71 %). This method detected all (100 %) nodules larger than 6 mm, 88 % of nodules in the range of >2–4 mm, and even 58 % of small nodules measuring only 1–2 mm. BLI identified a total of 40/82 lesions (sensitivity 49 %). The difference between PET and BLI was statistically significant at *p* < 0.05 (PET/BLI chi-square 8.2).

**Conclusions:**

PET was more sensitive than BLI for the detection of early peritoneal carcinomatosis in our mouse model. The sensitivity of BLI largely depended on the site of the lesions in relation to the imaging device.

## Background

Worldwide, gastric cancer is one of the most common malignancies. Despite recent advances in diagnosis and therapy, the prognosis of patients with gastric cancer remains poor [1]. Gastric cancer is often diagnosed in a locally advanced stage because of the lack of symptoms. Lymphatic and haematogenous spread may, however, be present even in the early stages [2]. Curative treatment is therefore possible in only approximately 50 % of patients. Even after potentially curative surgery (R0 resections), many patients develop recurrent disease, for which the treatment options are very limited [3].

One of the most severe conditions with initial gastric cancer spread or recurrent disease is peritoneal carcinomatosis, which has a median overall survival time of 3.1 months [4]. Sensitive detection and monitoring of peritoneal carcinomatosis is therefore essential for the initial staging and adequate treatment planning.

As a functional imaging modality, positron emission tomography (PET) with F^18^-flurodeoxyglucose (^18^ F-FDG) may represent an alternative to conventional imaging methods, such as CT and MRI, which are based mainly on the morphology. It has already been used for the staging and follow-up of various malignancies and has also been shown to provide additional information in the detection of peritoneal carcinomatosis [5].

Bioluminescence imaging (BLI) is a non-invasive imaging modality that has been developed over the last decade. It uses light emission produced by firefly (*Photinus pyralis*) luciferase in the presence of ATP and oxygen, following injection of the substrate D-luciferin. The emitted light is visualised by a cooled charge-coupled device (CCD) camera. This method has been successfully applied in many animal studies for the demonstration of tumour growth and therapeutic response [6].

To the best of our knowledge few data that would allow the evaluation of PET and BLI have yet been obtained in a single animal study. The aim of the present study was therefore to compare bioluminescence imaging and positron emission tomography for the early detection of peritoneal carcinomatosis in a mouse model.

## Methods

### Cell line

K. Yanagihara of the National Cancer Center Research Institute, Chuo-ku, Tokyo, kindly provided the human signet ring cell line HSC45-M2. It was isolated from the ascites and pleural effusion of a patient with a diffuse type of gastric cancer [7].

### Luciferase transfection

#### Plasmid construction

The coding sequence of firefly luciferase (fluc) was excised from pGL3 basic vector (Promega, Mannheim, Germany) by Hind III and Xba I digestion, blunted and ligated into the blunted Xho I site of the plasmid pcDNA3.1 (Invitrogen, Karlsruhe, Germany). The integrity of the cloned sequence (pcDNA3.1-fluc) was confirmed by automated DNA sequencing (GATC, Konstanz, Germany) using an ABI Prism 377 DNA sequencer (Applied Biosystems, Darmstadt, Germany).

#### Stable transfection of HSC cells

HSC cells were stably transfected with the expression vector pcDNA3.1-fLuc under the control of the cytomegalovirus promoter. Confluent cells were incubated in six-well plates with 0.8 g of pcDNA3.1-fluc, 20 μl Effectene and 6.4 μl enhancer per well, according to the manufacturer’s instructions (Qiagen, Hilden, Germany). Stable fluc-expressing HSC45-M2 cell clones were selected by treatment with 800 μg/ml Geneticin. The luciferase-transfected HSC45-M2 cells were grown in Dulbecco’s modified Eagle’s medium (DMEM) supplemented with 10 % fetal calf serum, 1 % penicillin/streptomycin (10,000 IU/10 mg/ml) and Geneticin (800 μg/ml), at 37 °C in a humidified atmosphere with 5 % CO2.

### Animal model

Sixteen 7-week-old female NMRI nude mice were given intraperitoneal (i.p.) inoculations of 1*10^7^ HSC45-M2-luc gastric cancer cells dissolved in 0.5 ml DMEM. The animals were divided into two groups (I and II) consisting of eight mice each. Imaging with PET and BLI was performed either on days 3 and 4 after inoculation (group I) or on days 6 and 7 (group II). One mouse died shortly after inoculation, leaving 15 mice available for analysis.

A mixture of 82 % saline, 10 % Ketavet [ketamine], and 8 % Rompun [xylazine] was used for anaesthesia during the imaging. After all the images had been obtained, the mice were sacrificed, and any lesions were documented and photographed. The tumour nodules found on post mortem examination served as the reference standard for evaluating the images. By choosing different points in time for the imaging, a broad spectrum of different nodule sizes was observed. Lesions were measured post mortem using a calliper, and the volume of tumours was calculated according to the formula length × width × height × 0.5. In face of some uncertainty remaining with regard to as to the true tumour volume in very small lesions, tumour nodules were categorised into relatively broad size ranges in order to avoid “pseudo-accuracy”. The tumour lesions were divided into five groups according to size (group 1 1–2 mm, group 2 >2–4 mm, group 3 >4–6 mm, group 4 >6-8 mm, group 5 >8–10 mm). Each nodule was also classed according to its specific location (e.g. liver surface or colonic region).

### Bioluminescence imaging

Bioluminescence imaging was performed with a cooled CCD camera coupled to a light intensifier unit (Hamamatsu). In vitro tests had been performed prior to the actual study in order to correlate cell number and semi-quantitative light signal gained by the CCD camera. These investigations were intended to document both transfection integrity and the linearity range of the imaging device (data not given) [8].

D-luciferin (sodium salt, SYNCHEM) was dissolved in sterile water to give a concentration of 15 mg/ml, filter sterilised and stored at −70 °C. The mice were injected i.p. with a mixture of 300 μl D-luciferin (15 mg/ml) and 200 μl anaesthetic (82 % saline, 10 % Ketavet, 8 % Rompun) 10 min before imaging. We tried to achieve the best possible intraperitoneal distribution of the substrate. The puncture was carried out on the linea alba, caudal to the umbilicus at an caudal angle between 30° and 45° to avoid intraintestinal injection and to protect other abdominal organs. Injections to the peritoneum were performed slowly in 0.5 ml medium with s-short subcutaneous needle and a 1 ml “insulin” syringe using a potentially saturating dose of D-luciferin. The distribution of the substrate was supported manually by mild massage and rotation of the mouse around the longitudinal axis.

For the imaging, mice were placed on a gel cushion in a dark box. Each mouse was recorded in the supine and lateral positions. First, photographs of the mice were taken in dimmed light. Then bioluminescent images were taken with an exposure time of 10 s. The dimmed light images were merged with the corresponding bioluminescent images and the light emissions transformed into pseudocolours. Hamamatsu Simple PCI software was used for image processing.

### PET

PET imaging was performed using a Phillips MOSAIC small animal scanner with a spatial resolution of 2.5 mm. The system is based on 14,456 individual GSO crystals with dimensions of 2 × 2 × 10 mm^3^, arranged in 52 rings of 278 crystals each. The port diameter was 19.7 cm. The transverse field of view was 12.8 cm, with an axial extent of 12.0 cm. The coincidence timing window was 12 ns; the standard energy window was between 410 and 665 keV.

The mice were given i.p. injections of 13 MBq of ^18^ F-FDG. The i.p. route was preferred over i.v. administration to prevent potential paravenous injections due to the very small calibre of the murine tail veins which could have significantly altered the biodistribution of ^18^ F-FDG in our tumour model. Fueger et al. performed a comparison of i.v. versus i.p. injection of ^18^ F-FDG and found a comparable distribution of the tracer for intravenous and intraperitoneal injection after 60 min. [9]. The prolonged uptake time of 3 h was chosen to allow for optimal background clearance. Anaesthetics were injected 10 min before imaging. The mice were placed in the prone position. Scan time was 15 min, using an isotropic voxel size of 1 mm and a matrix of 128 × 128 × 120 voxels. All images were reconstructed with a fully three-dimensional iterative reconstruction algorithm (3D–Ramla). Axial images were reformatted with coronal orientation using multiplanar reconstruction (MPR).

### Image interpretation

Two observers (JS and HW, with 20 and 16 years imaging experience, respectively) visually interpreted the PET and BLI images. The two readers were blinded with respect to the results of the post mortem examination. Any discrepancies were resolved by consensus. The imaging results were subsequently correlated with the post mortem findings. Lesions were classed as true positive, false positive or false negative. It was not possible to calculate a meaningful true negative fraction—either on the basis of the mice (because all animals had at least one tumour at post mortem) or on the basis of the lesions (because, in theory, there is an infinite number of negative tumour lesions per mouse). Determination of the diagnostic performance was therefore limited to the calculation of the sensitivity and the positive predictive value (PPV).

### Correlation of BLI signal and tumour volume post mortem

A correlation between BLI light signal and tumour volume post mortem (“in vivo”) was performed on basis of para-pancreatic lesions. The area was chosen for analysis because it was anatomically well defined, and the number of BLI positive lesions was comparatively high.

### Statistics

The sensitivity of BLI and PET in relation to the post mortem findings was calculated for mice as well as for lesions. The PPV was calculated on a lesion-by-lesion basis. The differences in terms of sensitivity and PPV were analysed statistically using the McNemar’s test for data related to the mice and the chi-square test for lesion-by-lesion data. The correlation of BLI signal and tumour volume post mortem (“in vivo”) was performed using the Spearman rank correlation coefficient. A value of *p* < 0.05 was taken to be statistically significant.

## Results

### Lesions and mice

All mice developed peritoneal carcinomatosis with similar patterns and extents by the time of death. Typical sites of tumour nodules were the surface of the liver, around the pancreas and the bowel mesentery. At post mortem, a total of 82 lesions were found in 15 mice.

All but one mouse had one or more positive lesions in PET imaging (sensitivity: 93 % (14/15)). Using BLI, all mice had at least one detectable lesion (sensitivity 100 % (15/15)). We recognised no unexpected low BLI signal in an individual mouse that would suggest accidental intraintestinal administration of D-luciferin. The difference between PET and BLI was not significant (*p* value 0.50).

### Accuracy according to the site of the lesion

Table 1 summarises the sensitivity of the imaging modalities in detecting lesions according to the anatomical site. Overall, 58 out of 82 lesions (sensitivity 71 %) were found with PET versus 40 out of 82 lesions (sensitivity 49 %) with BLI. The difference between PET and BLI was significant at *p* < 0.05 (chi-square 8.2152; *p* value 0.004154).

**Table 1:** Sensitivity for the detection of intraperitoneal lesions with PET and BLI depending on the site of the tumour

*n*	Site or region	Volume range	Sensitivity PET	Sensitivity BLI
		[mm^3^]	[%]	[%]
8	Injection site	0.3–9.0	63 (5/8)	100 (8/8)
2	Peritoneum	1.0–1.8	50 (1/2)	100 (2/2)
7	Diaphragm	0.1–1.8	14 (1/7)	0 (0/7)
13	Liver surface	0.5–3.0	85 (11/13)	8 (1/13)
1	Stomach	0.5	100 (1/1)	0 (0/1)
1	Duodenum	3.0	100 (1/1)	0 (0/1)
21	Jejunum	0.5–10.0	84 (16/21)	48 (10/21)
6	Colon	0.5–6.0	80 (4/6)	33 (2/6)
19	Pancreas	1.0–18	83 (15/19)	79 (15/19)
2	Uterus	1.8–3.0	100 (2/2)	100 (2/2)
2	Bladder	1.8–4.0	50 (1/2)	0 (0/2)
82	Total	0.1–18.0	74 (58/82)*	49 (40/82)*

The table shows the number of malignant lesions [*n*] with respect to different sites, the volume range and the sensitivities for PET and BLI. The difference between PET and BLI was significant at *p* < 0.05 (*chi-square 8.2152; *p* value 0.004154)

With PET, we obtained seven false-positive sites (in five mice) in the abdominal cavity and none with BLI. The positive predictive values were 0.89 % (58/65) for PET and 100 % (40/40) for BLI. The difference between PET and BLI (chi-square 4.6154; *p* value 0.031686) was significant at a level of *p* < 0.05.

### Accuracy according to the size of the lesion

**Fig. 1:**
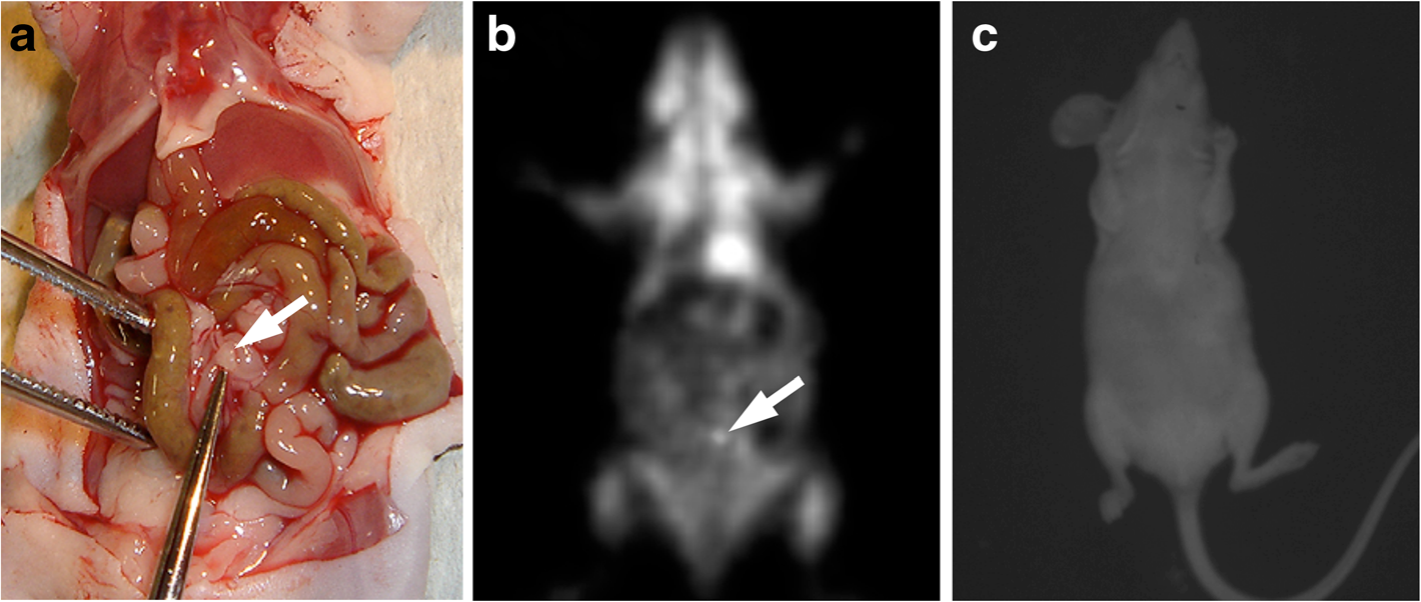
Small peritoneal lesion in the small bowel mesentery (*white arrow*) (**a**). The tumour measured 1.5 mm in diameter and was detected only with PET (**b**). The lesion was probably too deep in the abdominal cavity to be detected with BLI (**c**)

**Fig. 2:**
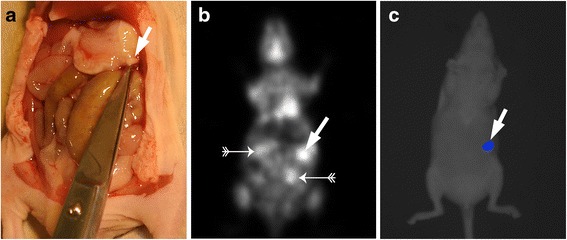
A 2.5 mm nodule in the para-pancreatic region found at post mortem (*white arrow*) (**a**). The lesion was detected easily with PET (**b**) and BLI (**c**) (*white arrows*). There were two false-positive sites on PET that could not be documented at post mortem (*small white arrows*) (**b**)

Lesion sizes ranged between 1 and 10 mm. Table 2 shows the detection rate for the imaging methods in relation to the size of the lesions. Overall, there was an obvious correlation between lesion size and the detection rate seen for all modalities. Despite its relatively limited spatial resolution compared with BLI, however, PET with ^18^ F-FDG detected even small nodules measuring 1–2 mm in 58 % of the cases (Fig. 1). PET detected all nodules larger than 6 mm. False-positive PET results could probably be attributed to non-specific bowel activity (Fig. 2).

**Table 2:** Sensitivity for the detection of intraperitoneal lesions by PET and BLI depending on tumour size

Size of the lesion	*n*	Sensitivity PET	Sensitivity BLI
		[%]	[%]
0–2 mm	50	58 (29/50)	40(20/50)
>2–4 mm	18	88 (15/17)	47 (8/17)
>4–6 mm	7	88 (7/8)	75 (6/8)
>6–8 mm	3	100 (3/3)	100 (3/3)
>8–10 mm	4	100 (4/4)	75 (3/4)
Total	82	71 (58/82)	49 (40/82)

The table shows the sensitivities for the detection of intraperitoneal lesions using PET and BLI with tumours of different sizes. There is a general correlation between lesion size and detection rate with both modalities

**Fig. 3:**
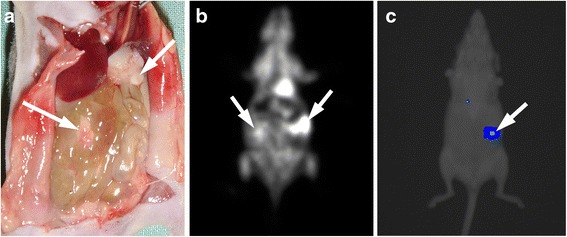
Post mortem finding of a 5.5-mm para-pancreatic lesion and a 4-mm lesion in small bowel mesentery (*white arrows*) (**a**). Both nodules were detected with PET (**b**). The lesion in small bowel mesentery remained undetected with BLI, most likely due to the site being covered by other bowel structures (**c**)

The detection of tumour nodules with BLI depended greatly on the site of the lesion. Even larger nodules located deep inside the peritoneal cavity were not delineated, most likely due to the limited depth penetration of the light (Fig. 3). However, small superficial nodules measuring 1–2 mm were successfully detected.

### Correlation of BLI signal and tumour volume post mortem

**Fig. 4:**
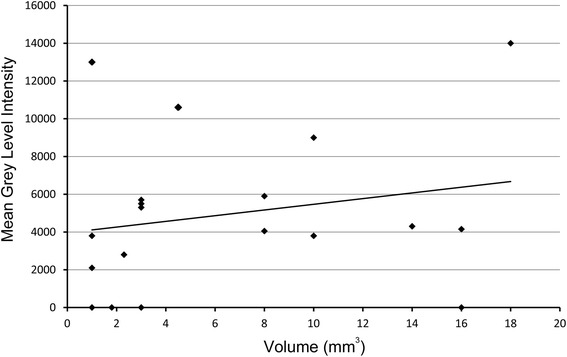
Correlation of BLI light signal (mean grey level intensity) and tumour volume in the para-pancreatic area determined post mortem. No significant correlation could be noted (Spearman rank correlation coefficient *r* = 0.2978, n.s.). A mild correlation is suggested by visual interpretation of the trend line. One larger lesion (16 mm^3^) remained undetected because it was covered by the left liver lobe

We found no significant correlation between BLI light signal and tumour volume post mortem (“in vivo”) for lesions in the para-pancreatic area. Even one larger lesion covered by the left liver lobe remained undetected (Fig. 4).

## Discussion

Early detection of peritoneal dissemination is essential for adequate treatment planning in the initial staging and follow-up of patients with gastric cancer. The diminishing role of surgical reassessment in treated patients has increased the reliance on cross-sectional imaging.

Accurate detection of peritoneal carcinomatosis by non-invasive means is a challenge in studies on both animals and humans. The peritoneum of the abdomen and pelvis provides a large surface area for microscopic seeding [10]. Tumour cells may grow on free peritoneal surfaces or invaginate the peritoneal folds over the mesentery or omentum, making some tumours extremely difficult to detect.

A number of imaging modalities for small animals evolved over the last decade and were successfully applied in numerous studies [8]. These modalities included also preclinical methods such as BLI. Functional imaging using PET was also introduced and scanners constructed for small animal use. These modalities, which differ significantly in temporal and spatial resolution, have inherently different technical requirements. To the best of our knowledge, however, few data comparing PET and BLI have yet been obtained in a single animal study. Our aim, therefore, was to evaluate the sensitivity and overall accuracy of these methods in detecting peritoneal carcinomatosis in a mouse model.

### PET

PET using ^18^ F-FDG was more sensitive than BLI in our study. In general, PET detected lesions independently of the site but with a clear correlation to the size of the lesions. As shown in Table 1, lesions on the peritoneal surface were detected in 60 % of cases (6/10), whereas only one out of seven lesions (14 %) was identified on the diaphragm. This low detection rate might be explained by the size of the nodules, since all undetected lesions measured less than 1 mm. This agrees with the results of Kondo et al. [11], who reported a limited sensitivity of ^18^ F-FDG PET for lesions smaller than 4 mm in their animal model.

We observed seven false-positive sites using PET, all of which were found in views of the bowel mesentery. These sites can probably be attributed to focal uptake of ^18^ F-FDG in the bowel, which is non-specific in nature and not related to tumour tissue. ^18^ F-FDG uses the same initial pathway as glucose and the uptake is therefore highly dependent on the presence of glucose transporters in the cell membrane. These transporters are present in tumour cells but also in many other tissues. ^18^ F-FDG is also taken up by inflammatory cells, which may explain false-positive findings in a clinical setting.

The lack of anatomical landmarks, relatively high costs and false-positive results due to focal uptake in non-malignant tissue might be regarded as potential limitations of PET. However, ^18^ F-FDG is still the most common tracer used in oncology for both clinical purposes and experimental setups. More specific tracers such as ^18^ F-FLT still need to be validated.

### Bioluminescence imaging

Bioluminescence imaging is a modality that perfectly suits the needs of small animal imaging. It is quick, quite easy to handle, and relatively inexpensive [12]. Bioluminescence imaging has been shown to be sensitive in various animal studies and has also been used to monitor tumour progression and relapse [13].

In our study, however, BLI was relatively insensitive compared with PET. This finding is, most likely, attributed mainly to light scattering and absorption (amount of tissue between cancer cells and CCD camera). When tumours are growing as a well-defined focal lesion close to the surface e.g. after flank injection, a good correlation between BLI signal and actual tumour mass is usually observed. This correlation is reduced in setups in which multiple lesions grow relatively dispersed in the abdomen, as it is the case in our intraperitoneal model [14]. The correlation between BLI light signal and tumour volume post mortem (“in vivo”) was not significant. BLI negative lesions resulted not only in a decrease in sensitivity but obviously have also substantial contribution to non-optimal correlation of light signal and tumour size (Fig. 4).

As might be expected, the sensitivity in the abdominal cavity depended on the site of the lesion. As shown in Table 1, all nodules attached to the peritoneum (*n* = 10) were detected by BLI. This high detection rate is probably explained by the superficial position of the lesions. Nodules shielded by dense structures or organs such as the liver frequently remained undetected. This might explain why none of the nodules on the diaphragm and only one lesion on the liver (1/13) could be seen.

It is important to notice, however, that the sensitivity of BLI is potentially dependent on various other factors, such as luciferase expression levels, transfection stability, oxygenation and tumour viability, as well as the performance and the setting of the camera. Some uncertainty may also remain with respect to a perfectly homogeneous distribution of the implanted tumour cells and the substrate D-luciferin in peritoneal cavity.

In principle, the substrate D-luciferin can be administered to animals using intraperitoneal (i.p.), subcutaneous (s.c.) and intravenous (i.v.) injections. After i.p. injection D-luciferin is absorbed by the peritoneum and reaches luciferase-expressing tissues mainly via bloodstream. In fact, variations in absorption rate through the peritoneum may distort the reproducibility of signal quantification [15]. A biodistribution study of radiolabeled D-luciferin demonstrated higher uptake in the gastrointestinal organs (pancreas and spleen) after i.p. injection than after i.v. injection. In addition, direct diffusion, other than the delivery via systemic circulation, may cause preferential distribution of D-luciferin in superficial intraperitoneal tumours close to the injection site [16]. However, the contribution of direct diffusion of the substrate to the overall signal is probably low owing to poor membrane permeability of D-luciferin [17]. Therefore, we think that i.p. administration may generally lead to overestimation of luciferase activity of IP tumours relative to extraabdominal tumours rather than causing “false” negative results in intraperitoneal lesions. However, s.c. injection of D-luciferin may be an alternative to i.p. injection for BLI of xenografts in nude mice particularly for tumours with weaker signals and when greater precision is required e.g. in signal validation studies [18].

Quantification of the BLI light signal may also be distorted due to technical reasons associated with CCD imaging. Larger tumours may show increased optical density and thus increased quenching of the signal as well as reduced availability of substrate to the tumour core compared to smaller tumours. This implies that tumour load may be underestimated when using BLI in vivo. The duration of light exposure to the camera is another potential factor influencing the sensitivity of the system. The exposure time is characterised by a trade-off between imaging depth and overexposure of superficial lesions. The parameter is therefore specific for the experimental setup and technical equipment. The exposure time in our study was derived from experience obtained in previous studies [8], which means that it was somewhat arbitrary in nature.

Although the correlation between light signal and tumour volume is not significant, changes in tumour mass relative to the initial BLI signal can still be accurately quantified when a uniform position of the animals towards the camera on the different imaging days is achieved. The BLI signal is, thereby, normalised to the baseline of each individual animal during the course of intervention [14].

### Limitations

Our study does have certain limitations. First, the numbers of animals and lesions undergoing imaging were relatively small. On the other hand, the pre-test probability of the presence of peritoneal tumour was quite high, because all mice had at least one tumour on post mortem examination. In consequence, our study did not address the true “sensitivity” of the methods used for detection but derived only relative sensitivities. For this reason, we cannot determine whether our findings could be extrapolated to a non-selected patient population or to other malignancies. Furthermore, the lack of a true negative fraction did not allow the specificity to be determined. The limited application of BLI in human studies is also obvious.

## Conclusions

PET was the more sensitive compared to BLI for detecting early peritoneal carcinomatosis in our mouse model. The sensitivity of BLI largely depended on the site of the lesions relative to the imaging device.

### Ethical approval

All applicable international, national and/or institutional guidelines for the care and the use of animals were followed.
